# Public response to the 2014 chemical spill in West Virginia: knowledge, opinions and behaviours

**DOI:** 10.1186/s12889-015-2134-2

**Published:** 2015-08-19

**Authors:** Elena Savoia, Michael A. Stoto, Rahul Gupta, Nasandra Wright, Kasisomayajula Viswanath

**Affiliations:** Department of Biostatistics and Division of Policy Translation and Leadership Development, Harvard T.H. Chan School of Public Health, 401 Park Drive, Boston, 02115 MA USA; Department of Health Systems Administration and Population Health, Georgetown University, 3700 Reservoir Road, NW Room 236, Washington, DC 20057-1107 USA; West Virginia Bureau for Public Health, 350 Capitol Street, Charleston, WV 25301 USA; Kanawha-Charleston Health Department, 108 Lee Street East, Charleston, WV 25301 USA; Department of Social and Behavioral Sciences, Harvard T.H. Chan School of Public Health, 677 Huntington Avenue, Boston, 02115 MA USA

## Abstract

**Background:**

On January 9^th^ 2014, a faulty storage tank leaked 10,000 gal of an industrial coal processing liquid into the Elk River in West Virginia (WV), contaminating the drinking water of the nine counties collectively known as the Kanawha Valley. The aim of this study was to 1) explore how and when people obtained information about the water contamination and 2) understand how individual and social factors such as socio-demographic characteristics, timing of information, trust in government, and risk perception influenced compliance with recommended behaviours and the public’s views on the need for environmental regulations.

**Methods:**

Between February 7–26, 2014, a survey was conducted of adult residents of West Virginia including geographic areas affected and non-affected by the chemical spill. The total population-based sample size was 690 and the survey was administered online. Descriptive statistics and multivariate statistical models were created to determine what factors influenced compliance and public opinions.

**Results:**

Findings from this study show that, during the 2014 West Virginia water crisis, information about water contamination spread quickly, as 73 % of survey respondents across the state and 89 % within the affected counties reported they heard about the incident the same day it occurred. Most people received the information promptly, understood what happened, and understood what to do to prevent exposure to the contaminant. The majority of respondents living in affected counties (70 %) followed the recommended behaviours. Among participants who voiced an opinion on the role of government in environmental regulations, the majority of respondents (54 %) reported there is “too little regulation.”

**Conclusion:**

Data from this study show that a higher perception of risk and timely receipt of information are associated with compliance with recommended behaviours, underlying the importance of releasing information to the public as quickly as possible during a crisis. This study also highlights the importance of coordinating risk communication activities beyond the area of the incident to assure public understanding of what measures are recommended, which are not and where.

## Background

On January 9^th^ 2014, a faulty storage tank leaked 10,000 gal of an industrial coal processing liquid into the Elk River in West Virginia (WV); the leak occurred upstream the main source of water for the company serving drinking water to the residents of nine counties collectively known as the Kanawha Valley. In the United States, chemicals have rarely been associated with waterborne disease outbreaks, for this reason the West Virginia water crisis can be described as a unique and interesting incident [1]. The January 2014 chemical contamination of the Elk River in West Virginia (WV) disrupted public water supply to thousands of homes, caused the closure of schools and businesses, and as a result, hundreds of people reported symptoms they associated with exposure to the contaminated water.

On January 9^th^, during the course of the day, state and local officials received several complaints of a licorice odor in the air and taste in the water due to the presence of the chemical [2–4]. At 6 pm the day of the leak, Governor Earl Ray Tomblin appeared on television and issued a “Do Not Use” order that was unprecedented in scale or scope. As a result, approximately 300,000 residents (16 % of the population of West Virginia), living in the nine counties affected by the spill, were told that their tap water was not safe for “drinking cooking, washing, or bathing” and to be aware of signs and symptoms of exposure to the chemical such as rashes, nausea, vomiting, and wheezing [5]. Besides local television news, state and local officials used many other channels of communication, including radio, social media and town halls. A description of the timeline of the response and specific challenges experienced by the responders in coordinating communication efforts have been described elsewhere [6].

At the time of the incident, very little toxicological information was available on the main chemical contained in the liquid: 4 -methylcyclohexane methanol (crude MCHM). According to the producer, MCHM is harmful if swallowed, causes skin and eye irritation, and at elevated temperatures can cause irritation of the eyes and respiratory tract [7]. Potential carcinogenic effects were investigated after the incident showing DNA damage and need for further carcinogenic evaluations [8].

In the week following the spill the water distribution system was flushed and residents of the affected areas were advised to flush their plumbing systems using a stepwise protocol [9]. However, after the “Do Not Use” order was lifted the odor remained and questions about the health effects of MCHM persisted [6].

The cultural, political, and economic context of the Kanawha Valley created a unique case in which to study the population’s exposure to information, their knowledge, behaviours and reactions to this emergency. For nearly a century, the Kanawha Valley has been home to the largest concentration of chemical plants in the United States [10]. Valley residents are not unfamiliar with the risk of chemical spills, environmental agencies receive several calls on a weekly basis on potential spills from industries working in the area, [6] but in this circumstance little was known about the health risks of the chemical product that spilled in the water and the persistent odor and taste generated concern in the population. For decades, residents in the valley have participated in long running debates over environmental oversight of the coal and chemical industries, the foundation of West Virginia’s economy.

In times of low risk as well as in times of emergencies, in West Virginia as well as in any other area, information about the potential threat of chemicals or pollutants circulates widely from both official and unofficial sources and exposure to circulating information may influence or not behaviours [11]. Under conditions of uncertainty on the extent of exposure to a particular product and its impact on health, risk communication becomes a challenging dynamic process [12] in which the public has a myriad of opportunities to seek, assimilate, and act or do not act on the information received and to accept or contest official opinion and advice [13]. More specifically, during large scale emergencies, several studies have shown that risk communication may fail to reach intended communities particularly those most at risk [11–17].

The aim of this study is to 1) explore how and when people obtained information about the water contamination and 2) understand how individual and social factors such as socio-demographic characteristics, timing of information, trust in government, and risk perception influenced compliance with recommended behaviours and the public’s views on the need for environmental regulations.

## Methods

Between February 7–26, 2014, a survey was conducted of adult residents of West Virginia including geographic areas affected and non-affected by the chemical spill. The total population-based sample size was 690; 145 respondents were drawn from the local sample of Knowledge Networks’ KnowledgePanel®, a survey system with an address-based sample frame that provides non-Internet capable households with a laptop computer and free Internet access. The remaining participants (545) were recruited solely for the purpose of this survey (opt-in-panel), using the KnowledgePanel calibration technique [17] with an over-sample of residents of low socio-economic position (SEP). Base weights and panel post-stratification weights were included with the sampling technique. We sought to oversample individuals with low SEP so we would have a large enough sample to identify potential barriers in receiving and understanding public health messages due to low literacy levels and/or limited access to some channels of communication. Participants were eligible to win prizes drawn through monthly sweepstakes.

### Survey design

The questions for this survey were adapted from a survey implemented in the aftermath of the 2010 Boston Water Crisis [18] and from polls conducted by Gallup® [19]. The survey covered knowledge and adoption of preventive behaviours, perception of risk, use of information channels, and the public’s views and reactions on the role of government. Pilot testing of the survey was conducted on 45 individuals prior to its fielding. The study was approved by the Harvard T.H. Chan Institutional Review Board (IRB).

### Measures

#### Independent variables

Independent variables were originally selected on the basis of substantive and theoretical relevance in accordance with the “*Structural Influence Model of Communication in Public Health Emergency Preparedness*” [11–16, 18]. This heuristic model focuses on the role of communication in explaining the relationship between social determinants and health outcomes, and has been successfully used to examine communications during the 2010 Boston water crisis [18]. Six types of independent variables were included: area of residence (living or not in one of the affected counties),^1^ social determinants (education, income, savings), perceived risk, trust in government, whether and when the respondent learned about the emergency and demographic variables (gender, age). More details on how each variable was defined and categorized are provided in Table 1.

**Table 1:** Definition of variables

Age	a) 18–29, b) 30–44, c) 45–59 and d) ≥ 60
Education	a) < high school, b) high school, c) some college and d) bachelor degree or higher
Household income in the past 12 months	a) ≤ $14,999, b) $15,000–$34,999, c) $35,000–$59,999, d) $60,000–$99,999 and e) ≥ $100,000.
Savings - *If you lost your current source of income (your paycheck, public assistance, or other forms of income) how long could you continue to live at your current address and standard of living?*	a) 0 = less than 2 months, b) 1 = 3 months-one year and c) 2 = more than one year.
Timeliness of information - *When did you first learn about the water crisis?*	a)1 = the same day the water contamination was reported (Thursday January 9) = 1 b) one or more days after =0.
Perceived risk -*To the best of your knowledge, how likely was it for someone who lived in the affected areas to get sick by drinking tap water during the water crisis?*	a) 0 = very unlikely or unlikely, b) 1 = somewhat likely and c) 2 = very likely.
Trust in government -*Would you say that the government generally is?*	a) 0 = run by few people looking for their own interests and b) 1 = run for the benefit of all.
Knowledge of recommended behaviors -*To the best of your knowledge what actions were recommended in the areas affected by the water crisis?* This question had correct response options describing recommended behaviors (e.g. drinking bottled water, avoiding drinking tap water, avoiding taking showers or bathing with tap water, avoiding using tap water to prepare baby formula) and non-correct options recommended behaviors (e.g. drinking water only after boiling it, drinking filtered tap water, cooking with boiled or disinfected water, washing dishes with boiled or disinfected water and brushing teeth with boiled or disinfected water).	a) 1 = the respondent checked at least one recommended behavior and did not check any non-recommended behavior b) 0 = the respondent did not check any of the recommended behaviors or checked at least one non-recommended behavior.
Behavioral Compliance	a)Recommended behaviors (e.g. drank only bottled water, cooked with only bottled water, washed dishes with only bottled water, brushed teeth with only bottled water, avoided taking showers or bathing with tap water, avoided tap water from coming in contact with skin, avoided using tap water for washing clothes),
	b)Non-recommended behaviors (e.g. drank water after boiling it, cooked with only boiled or disinfected water, washed dishes with only boiled or disinfected water, brushed teeth only with boiled or disinfected water), and
	c)Non-recommended behaviors geographically specific to affected counties (drank tap water and followed no recommended behavior).
	1 = the respondent checked at least one recommended behavior and did not check any non-recommended behavior or 0 = the respondent did not check any of the recommended behaviors or checked at least one non-recommended behavior.
	Flushing compliance: After learning the water crisis was over, did you flush a) cold water faucets for at least 5 min?, b) warm water faucets for at least 15 min?, c) appliances (i.e. ice maker, dish washer). Answer options to this question were Yes/No.

#### Dependent variables

A scoring system was devised to create two dependent variables: 1) compliance with recommended behaviours and 2) views on government’s role and priorities. To measure *compliance with recommended behaviours,* responses to the following question were analyzed: *In response to news reports of the water crisis, did you do any of the following?* The response options included recommended and, non-recommended behaviours listed in Table 1. Based on these responses we created a count variable ranging from 0 (did not follow any recommended behaviour, followed a non- recommended behaviour or followed a combination of recommended and not recommended behaviours) to 1–7 (maximum number of recommended behaviours).

Once the water was determined to be appropriate for use, people in the affected counties were recommended to flush faucets and appliances to eliminate any residual contaminants. Compliance with this procedure was assessed through responses to three questions as described in Table 1.

To measure *opinion on government’s role and priorities*, two variables were generated using data from the following questions: 1) *In general do you think there is too much, too little, or about the right amount of government regulation on the environment?* and 2) *Do you think it should be a high, medium or low priority for the government to regulate the way major chemical and coal industries do business or the government should not address this issue?* Separate ordinal variables were created where responses were coded as 0 = too much, 1 = right amount and 2 = too little and 0 = government should not address this issue or it should be a low priority, 1 = a medium priority and 2 = a high priority.

### Statistical analyses

Study specific post stratification weights based on gender, age, metropolitan area, race/ethnicity, education and income were created using the most recent Census Bureau Current Population Survey to adjust for non-coverage and non-response biases. Descriptive statistics were calculated showing the frequency of both the dependent and independent variables. For count variables, negative binomial regression was used in bivariate and multivariate models after rejecting the hypothesis of equivalence in the mean and variance of the count. For ordinal variables, ordered logistic regression was applied using Stata’s *ologit* command and the parallel regression assumption was tested by means of the *Brant* test when needed. In all analyses study specific weights were included to test for bivariate associations between each predictor and the two dependent variables. A *P*-value of ≤ 0.25 was used as a cut-off to include the variables in the model*.* In doing so, the following variables were dropped from the multivariate analysis of compliance: age, gender, education, savings, and trust in government. For the analysis of opinions on the role of government in regulating the environment the following variables were dropped: education and savings. Interactions were tested between area of residence (within or outside the nine affected counties) and the two following variables: timeliness of information and knowledge of recommended behaviours. Analyses were performed using Stata statistical software, release 13, College Station, TX.

## Results

### Sample characteristics

The overall response rate was 86 %, with 592 individuals of the 690 sampled beginning the survey. Of these, 464 (67 % of the original sample) completed the entire survey, and their responses were included in the final analyses. Of the 464 respondents, 129 (27.8 %) lived in one of the nine counties affected by the crisis, while the remaining 335 (72.1 %) lived in West Virginia but outside the affected counties. Data from respondents from all counties were analyzed and differences were investigated between respondents from the affected areas and the rest of the state. The proportion of respondents living in the affected counties did not vary between the KnowledgePanel® and opt-in panel subsamples (27 % versus 28 %). Socio-demographic characteristics of those living in and outside the affected counties are shown in Table 2. The raw data (before weighting) show that the subsample of people living in the affected counties have a higher level of education compared to those living outside this area, but between the two groups there were no statistically significant differences in any of the socio-demographic variables including education. Despite intentional oversampling in the opt-in sample, when the sample characteristics were compared to the Census data our sample was found to underrepresent the category of educational level “less than high school.”

**Table 2:** Sample characteristics versus U.S. Census Bureau (American Community Survey 2009–2013)

Variable	Total sample n (%)	Census	Living in the affected counties n (%)	Census	Living outside the affected counties n (%)	Census
Gender (*n* = 464)						
Male	220 (47.4)	49 %	58 (44.6)	49 %	161 (48.3)	49 %
Female	244 (52.6)	51 %	71 (55.4)	51 %	173 (51.7)	51 %
Age (*n* = 463)						
18–24	29 (6.3)	6 % [20–24]	9 (7.0)	6 % [20–24]	20 (6.0)	7 % [20–24]
25–34	69 (14.9)	12 %	17 (13.2)	12 %	52 (15.5)	12 %
35–44	63 (13.6)	13 %	22 (17.1)	13 %	41 (12.3)	13 %
45–54	106 (22.8)	15 %	27 (20.9)	15 %	79 (23.7)	15 %
55–64	129 (27.8)	14 %	36 (27.9)	8 %	93 (27.8)	17 %
65–74	57 (12.3)	9 %	17 (13.1)	16 %	40 (12.0)	7 %
75+	10 (2.2)	7 %	1 (0.8)	7 %	9 (2.7)	7 %
Education						
(*n* = 463)	33 (7.2)	17 %	7 (5.2)	16 %	26 (7.9)	17 %
< High school	221 (47.7)	41 %	47 (36.4)	39 %	172 (51.5)	42 %
High school	104 (22.5)	24 %	38 (29.6)	25 %	67 (20.1)	24 %
Some college Bachelor or more	105 (22.6)	18 %	37 (28.8)	20 %	69 (20.5)	17 %
Income (*n* = 450)						
<$10,000	26 (5.6)	10 %	7 (5.4)	10 %	19 (5.9)	10 %
$10,000–$24,999	48 (10.4)	22 %	19 (14.7)	23 %	29 (9.0)	22 %
$25,000–$49.999	124 (26.7)	27 %	29 (22.5)	28 %	95 (29.3)	26 %
$50,000–$74.999	98 (21.1)	18 %	25 (19.4)	12 %	73 (22.5)	20 %
$75,000–$99.999	82 (17.7)	10 %	22 (17.1)	12 %	60 (18.5)	10 %
$100,000–$199,999	63(13.6)[$100,000–$174,999]	11 %	21(16.3)[$100,000–$174,999]	12 %	42(13.0)[$100,000–$174,999]	10 %
>$200,000	9 (1.9)	2 %	3 (2.3)	3 %	6 (1.8)	2 %

#### Risk perception

Fifty-six percent of respondents reported that “*getting sick from drinking tap water”* was very likely, with no difference between those living in the affected counties and those living outside this area (50 % and 58 % respectively Binomial *P*-value > 0.05). Respondents who reported a household income greater than $100,000 were the least likely to think that drinking tap water would cause sickness when compared to respondents in lower income categories. For example, those reporting an income of < $15,000 were twice as likely to report drinking water as risky compared to those with an income of > $100,000 (OR = 2.37, 95 % C.I. 1.07–5.22). Similarly, respondents who reported they could not afford their current standard of living for more than two months if income was lost were almost twice as likely (OR = 1.9, 95 % C.I. 1.3–3.05) to think that drinking the water would cause sickness compared to respondents that could afford their standard of living for over a year. Most of the respondents (56 %) agreed or strongly agreed with the following statement: “*Chemical or oil spills in the water are happening all the time every day”*.

#### Timeliness of information

Seventy-three percent of respondents answered that they heard about the water crisis on the same day the water contamination was reported (January 9). The cumulative percentages of those who heard about the crisis rose to 93 % by January 10, and 100 % by January 12. People living in the affected counties were 3.7 times more likely to have heard the news on the day the spill occurred compared to people living outside this area [OR = 3.7 95 % C.I. 1.9–7.2].

#### Source of information

**Fig. 1:**
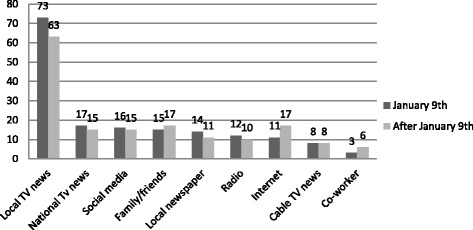
Percentage of respondents hearing about the water crisis: first source of information and date

Independently of when they received the news, most people (70 %) first heard about the spill from local TV. The Internet, family, friends and co-workers were the sources of information more frequently reported as first source the news was heard from. See Fig. 1 for a description of source of information by date the information was received.

#### Trust in government

When asked the question “*Would you say that the government generally is …?”* 77 % of respondents agreed with the statement “*Run by few people looking for their own interests.”* This percentage ranged from 86 % for those living in the affected counties to 75 % for those living outside this area*;* 14 % agreed with the statement *“Run for the benefit of all”* (10 % in the affected counties and 16 % outside this area) and 8 % checked the option “*Other, please specify”.* Among this 8 %, the majority (65 %) reported a negative comment denoting a lack of trust, such as “*Run for the benefit of large lobby groups*” or “*ineffective,*” while 22 % reported mixed opinions such as “*half and half”* or *“not all one category,”* and 13 % were unsure. Trust in government was subsequently used as an independent variable in the multivariate models.

#### Knowledge of recommended behaviours

The percentage of respondents who did not know about any recommended behaviour was low among residents of the nine affected counties; 89 % of them were able to report at least one recommended behaviour. In terms of non-recommended behaviours, “*washing dishes with boiled or disinfected water”* was the most frequently reported, with 9 % of residents in the affected counties and 20 % of residents in the non-affected counties reporting it as a beneficial action. Other behaviours incorrectly identified as “recommended” include: brushing teeth with boiled or disinfected water (7 % affected and 18 % non-affected counties), cooking with boiled or disinfected water (6 % affected and 17 % non-affected counties), and drinking water after boiling it (2 % affected and 4 % non-affected counties). Knowledge of recommended behaviours was not associated with any of the socio-demographic factors, area of residence, or any other independent variables listed above.

#### Compliance with recommended behaviours

The main recommendation was to abstain from drinking tap water, and 69 % of respondents living in the affected counties complied. There was a positive association between compliance with this recommended behaviour and perception of risk, measured as the belief that drinking the tap water will cause sickness [Pearson chi-squared *p*-value = 0.02]. Overall, 74 % of respondents living in the affected counties were compliant with at least one preventive measure. People living in an affected county were 5.7 times more likely to follow a recommended behaviour than those living outside this area [95 % C.I. 3.8–8.6]. Interestingly, outside the affected counties 22 % of respondents followed at least one of the recommended measures, even though not necessary. Residents who received information the same day the emergency occurred were 2.3 times more likely to follow a recommended behaviour [OR = 2.3 95 % C.I. 1.2–4.5]. Similarly, people reporting a belief that drinking tap water in the affected areas was very likely to cause sickness were 2.4 times more likely to follow a recommended behaviour [OR = 2.4 95 % C.I. 1.1–5] when compared to those who thought that it was unlikely or very unlikely to make people sick. See Table 3.

**Table 3:** Determinants of behavioral compliance

Independent Variables	Single Predictors Models	Ordinal Logistic Regression Models		
	IRR (95 % C.I.)	Model 1	Model 2	Model 3
Living in the affected counties	5.8 [3.9–8.5]	6 [4–9]	6 [4–9.2]	5.7 [3.8–8.6]
Income				
$15,000–$34,999 vs < $15,000	0.7 [0.3–1.1]	0.8 [0.3–1.9]	0.8 [0.4–1.7]	0.8 [0.4–1.6]
$35,000–$59,999 vs < $15,000	0.6 [0.3–1.1]	0.5 [0.2–1.2]	0.6 [0.3–1.3]	0.6 [0.3–1.4]
$60,000–$100,000 vs < $15,000	0.8 [0.4–1.4]	0.7 [0.3–1.5]	0.8 [0.3–1.7]	0.8 [0.4–1.7]
>$100,000 vs < $15,000	0.6 [0.3–1.2]	0.4 [0.2–1.1]	0.5 [0.2–1.3]	0.5 [0.2–1.2]
Timing of information	2.8 [1.4–5.5]		2.3 [1.2–4.6]	2.3 [1.2–4.5]
Risk perception				
•Somewhat likely versus unlikely or very unlikely	1.3 [0.7–2.5]		1.1 [0.5–2.5]	1.2 [0.5–2.5]
•Very likely versus unlikely or very unlikely	2 [1.1–3.7]		2.3 [1.1–4.8]	2.4 [1.1–5]
Knowledge of recommended behaviors				1.8 [1–3.2]

In the affected counties, the recommended procedure to flush cold water faucets for 5 min was followed by 86 % of respondents, flushing warm water faucets by 68 %, and flushing appliances by 90 %. Outside the affected areas, 14 % of respondents flushed their cold and warm water faucets and 37 % flushed their appliances.

#### Views on government’s role and priorities

Among participants who voiced an opinion on the role of government in regulating the environment (82 %), the majority of them (54 %) reported there is “too little regulation.” This percentage was much higher among those living in the affected counties (74 %) compared to those living outside this area (48 %). Twenty percent of all respondents reported that the amount of regulation is adequate (11 % in affected counties and 26 % outside), and 23 % said there is too much regulation (15 % in affected counties and 26 % outside).

The multivariate analysis (Table 4) shows that respondents in the affected counties were 2.6 times more likely [OR = 2.6 95 % C.I. 1.5–4.5] to think that there is too little government regulation when compared to respondents living outside this area. Gender was also associated with this opinion, with women being 1.9 times more likely [OR = 1.9 95 % C.I. 1.2–2.9] to think there is too little regulation when compared to men. Similarly, higher risk perception was associated with this opinion. People reporting the highest category of risk perception (very likely to get sick if drinking tap water) in the affected areas were 2.4 times more likely [OR = 2.4 95 % C.I. 1.2–5.1] to think there is too little regulation compared to those that believed it was unlikely or very unlikely they would get sick from drinking the tap water.

**Table 4:** Determinants of opinion on the existing amount of government regulations on the environment (too little, right amount, too much)

Independent Variables	Single Predictor Models	Multiple Negative Binomial Regression Models		
	OR (95 % C.I.)	Model 1	Model 2	Model 3
Living in the affected counties	3.4 [1.5–5]	2.8 [1.7–4.6]	2.9 [1.7–4.7]	2.6 [1.5–4.5]
Gender	1.8 [1.1–2.9]	1.9 [1.2–2.9]	1.8 [1.2–2.9]	1.9 [1.2–2.9]
Income				
$15,000–$34,999 vs < $15,000	0.3 [0.2–1.6]	0.5 [0.2–1.3]	0.4 [0.2–1.1]	0.4 [0.2–1.1]
$35,000–$59,999 vs < $15,000	0.6 [0.3–1.5]	0.8 [0.3–1.8]	0.7 [0.3–1.6]	0.7 [0.3–1.6]
$60,000–$100,000 vs < $15,000	0.5 [0.2–1.3]	0.6 [0.3–1.4]	0.6 [0.2–1.3]	0.5 [0.2–1.2]
>$100,000 vs < $15,000	0.4 [0.2–1.1]	0.4 [0.2–1]	0.4 [0.2–1]	0.4 [0.2–1.1]
Timing of information	0.7 [0.4–1.2]		0.7 [0.4–1.2]	0.8 [0.5–1.3]
Risk perception				
•Somewhat likely versus unlikely or very unlikely	2.7 [1.2–6.1]		1.8 [0.9–3.6]	1.9 [0.9–4.2]
•Very likely versus unlikely or very unlikely	2.4 [1.1–5]		2.1 [1–4.2]	2.4 [1.2–5.1]
Trust in government	0.7 [0.4–1.1]			0.8 [0.5–1.4]
Knowledge of recommended behaviors	1.1 [0.7–1.8]			1 [0.6–1.6]

When respondents were asked their opinion on whether regulating major chemical and coal industries should be a high, medium, or low priority for government, the majority (55 %) reported that it should be a high priority, 31 % reported it should be a medium priority, 6 % a low priority and 7 % that the government should not address this issue. Finally, respondents were asked how much they agreed or disagreed with the following statement “*In this region chemical or oil spills are an acceptable compromise to have a job”.* The majority (57 %) reported they disagreed or strongly disagreed with this statement independently of their area of residence.

## Discussion

Findings from this study show that, during the 2014 West Virginia water crisis, information about water contamination spread quickly, as 73 % of survey respondents across the state and 89 % within the affected counties reported they heard about the incident the same day it occurred. Most people received the information promptly, understood what happened, and understood what to do to prevent exposure to the contaminant. The majority of respondents living in the affected counties (694 %) were compliant with the main recommendation of abstaining from drinking the tap water. Thirty-seven percent living outside this area also avoided drinking the tap water during the crisis even if not recommended, one possibility explaining this action could be a belief that the contaminant will eventually reach the water supply of other communities and highlight a need for coordinating risk communication activities beyond solely affected areas.

Respondents in the affected counties learned about the crisis sooner and were more likely to follow the recommended behaviours. In contrast, those in the non-affected counties, more frequently erroneously believed that boiling or disinfecting the water was a recommended behaviour. Respondents in the non-affected areas (who were not exposed to the contaminated water), on the other hand, were almost as likely as those in the affected areas to believe that getting sick from drinking the water was very likely. This suggests that risk communication efforts worked reasonably well for the affected population, but caused confusion in other parts of the state.

Data from this study clearly show that a higher perception of risk and timely receipt of information are associated with compliance with recommended behaviours, underlying the importance of releasing information to the public as quickly as possible during a crisis. In contrast to previous studies we did not find a difference in compliance across socioeconomic levels [20–24]. Our results on the association between risk perception and compliance are consisted with previous studies referring to “*Protection Motivation Theory”* (PMT) as a potentially useful framework for understanding public response or intention to respond to recommended health behaviours [25]. In our study we emphasize the importance of observing actual behaviours after a crisis and to include in such observations factors related to the response of the public health system (i.e. time of release of information to the public) to contextualize and better explain public behaviours and inform the response to future crisis. During this event, proactive public health strategies and a saturation of media coverage likely resulted in a wide diffusion of information, a study on the relationship between the use of specific channels of information by the public and timing of the receipt of information was beyond the purpose of this paper.

Furthermore, the literature describes trust and credibility as critical components of effective risk communication [26–30]. In this study we limited our observation of “trust” to generic questions on “trust in government” which resulted not to be associated with compliance with recommended behaviours or opinions on the role of government in regulating the environment. We recognize that trust is a complex construct and that our study does not adequately describe such construct. However, this crisis seems to have exceeded the public’s tolerance for lack of government oversight on environmental regulations. The fact that we found a difference in such opinions between residents in the affected versus non-affected counties support our hypothesis that the experience of the incident influenced their opinion on the need for regulations; as shown by 74 % of survey respondents, living in the affected areas, reporting that there is “too little … government regulation” on the environment, a view also found to be disproportionately held by women and those with a high perception of risk. Such findings may be of potential interest to the policy maker.

The main limitation of this study was an underrepresentation of respondents of low socio-economic position and low educational level, which might have limited our ability to describe behaviours and opinions among this group. However all statistical analysis took socio-demographics into consideration, by including study specific post-stratification weights.

The use of a cross sectional study limited the ability to determine public opinions and behaviours prior to the emergency. However the fact that respondents living in the affected counties were more likely to have an opinion on the need for regulations suggests that the experience of living through the emergency influenced such opinion. That is, the proximity of the threat influenced their risk perceptions, and subsequent behaviours.

## Conclusion

Findings from this study show that during a crisis communicating to the public in a timely manner affects compliance with recommended behaviours, and that risk-perceptions are associated with compliance. This study also highlights the importance of coordinating risk communication activities beyond the area of the incident to assure public understanding of what measures are recommended, which are not and where. Finally, results may be of interest to the policy maker in light of the finding that people that experienced the crisis had stronger opinions on the role of government in regulating the environment compared to those living in non-affected areas.

## Abbreviation

MCHM: 4 -methylcyclohexane methanol

## Footnotes

1 Boone, Cabell, Clay, Jackson, Kanawha [which includes the city of Charleston], Lincoln, Logan, Putnam or Roane.
